# The iBRA-NET localization study: international IDEAL 2a/2b multicentre cohort study comparing the safety and effectiveness of wire- and radar-guided localization for impalpable breast lesions

**DOI:** 10.1093/bjsopen/zrag108

**Published:** 2026-09-10

**Authors:** Rajiv V Dave, Jenna Morgan, Simon Lowes, Robert Milligan, Rachel Foster, Emma Barrett, Amtul R Carmichael, Suzanne Elgammal, Edward St. John, Chris Holcombe, Yazan Masannat, Shelley Potter, Suzanne Krizak, Vidya Raghavan, James Harvey, the iBRA-NET Localisation Study collaborative, A Pradeep, S Muktar, L Martin, J Noronha, K Mallalieu, S Ledwidge, J Tan, S Khan, D Debnath, P McAleese, A McKenna, E Afify, L Ballance, P O’Leary, F Meybodi, I Oikonomou, C Liyang Wang, Mok, C Hollywood, C Mak, S Naz, K Kolar, C Roshanlall, R Swan, M Gasparri, M Walker, C Ríos Gozálvez, C Sitges, A Catanese, C Sitges, A Mariscal, J Sánchez-Méndez, E Khalifa, A Nagarajakumar, I Giono, E de Sousa, J Henderson, L Swainbank, I Sallam, R Lefroy, G Babu, E Macaskill, K McNeil, S Vural, A Aber, N Emmanuel, L Gharbawi, A Taniskidi, J Ragunathan, K Griffith, B Smith, A Hakim, A Bassi, R Burrows, L Mackay, H Fatayer, S D’Costa, M Abdelhadi, I Whitehead, G Ng, S Mylvaganam, E Rahman, I Sallam, M Shaaban, A Michaelides, M Khatib, N Ditsch, A Hamad, E Mohamed, O Alaamer

**Affiliations:** The Nightingale Breast Cancer Centre, Wythenshawe Hospital, Manchester University NHS Foundation Trust, Manchester, UK; Faculty of Biology, Medicine and Health, University of Manchester, Manchester, UK; Division of Clinical Medicine, University of Sheffield Medical School, Sheffield, UK; Jasmine Centre, Doncaster and Bassetlaw Teaching Hospitals NHS Trust, Doncaster, UK; Northern Centre for Breast Research, Gateshead, UK; Translational and Clinical Research Institute, Newcastle University, Newcastle upon Tyne, UK; Northern Centre for Breast Research, Gateshead, UK; The Nightingale Breast Cancer Centre, Wythenshawe Hospital, Manchester University NHS Foundation Trust, Manchester, UK; Department of Medical Statistics, Manchester University Hospitals NHS Foundation Trust, Wythenshawe Hospital, Manchester University NHS Foundation Trust, Manchester, UK; University Hospital of Derby and Burton NHS Foundation Trust, Queens Hospital, Burton upon Trent, UK; University Hospital Crosshouse, NHS Ayrshire and Arran, Kilmarnock, UK; Department of Breast Services, Portsmouth Hospitals University NHS Trust, Portsmouth, UK; Faculty of Science and Health, University of Portsmouth, Portsmouth, UK; Breast Unit, Royal Liverpool University Hospital, Liverpool, UK; Department of Breast Services, Broomfield Hospital, Chelmsford, UK; Faculty of Health, Medicine and Social Care, Anglia Ruskin University, Cambridge, UK; Translational Health Sciences, Bristol Medical School, Bristol, UK; Bristol Breast Care Centre, North Bristol NHS Trust, Bristol, UK; The Nightingale Breast Cancer Centre, Wythenshawe Hospital, Manchester University NHS Foundation Trust, Manchester, UK; Breast Care Service, Royal Wolverhampton NHS Trust, Wolverhampton, UK; The Nightingale Breast Cancer Centre, Wythenshawe Hospital, Manchester University NHS Foundation Trust, Manchester, UK; Faculty of Biology, Medicine and Health, University of Manchester, Manchester, UK

**Keywords:** wide local excision, surgical margins, re-excision, operative workflow, non-palpable lesions

## Abstract

**Background:**

Wire-guided localizations have been the historical standard for breast lesion localization; however, they have logistical constraints and can dislodge in the preoperative period. Radar-guided localization has the potential to uncouple localization from the day of surgery, but the technique needs to be evaluated in a direct comparison with wire-guided localization to ensure efficacy and safety.

**Methods:**

The iBRA-NET localization study is a prospective platform IDEAL 2a/2b study comparing wire-guided localization with new generation localization devices. This arm describes SCOUT® radar localization for impalpable breast lesions performed between June 2021 and December 2024, compared with a historical cohort of wire-guided localization performed between August 2018 and August 2020. Primary outcome was identification of the index lesion in the excision specimen. Secondary endpoints included safety (complications), margin re-excision, magnetic resonance imaging bloom size, and specimen weight.

**Results:**

Data were accrued from 1297 radar localized patients in 27 units. Index lesion identification was 95.2% in radar-guided excisions *versus* 99.1% in wire-guided excisions (*P* = 0.001). In a sensitivity analysis excluding patients receiving neoadjuvant chemotherapy, identification rates were 98.9% *versus* 99.6% (*P* = 0.114). For a subset of patients having a simple lumpectomy for a unifocal, unilateral breast lesion with no neoadjuvant treatment (1627), positive margins were seen in the main excision specimen in 1003 (15.0%) wire-guided *versus* 624 (9.0%) radar-guided excisions (*P* < 0.001). Re-excision was required in 131 (13.2%) wire-guided *versus* 91 (14.7%) radar-guided excisions (*P* = 0.442). There was a significant difference in specimen weight/size^2^ (0.138 g/mm^2^ wire-guided localization *versus* 0.182 g/mm^2^ radar; *P* < 0.001). The median start time for surgery was earlier in the day for radar-guided localizations compared with wire-guided (12:27 *versus* 13:40; *P* < 0.001). All radar placements in axillary nodes (13) led to successful nodal retrieval.

**Conclusion:**

SCOUT® radar localizations are a safe and effective localization technique in both the breast and axilla, with outcomes comparable to wires. There were demonstrable logistical advantages in operative start time when using a radar reflector.

## Teaser

This article is the latest arm of the iBRA-NET localization study and reports on the efficacy and safety of radar-based breast localization techniques compared with wire guidance. The study confirms that radar localization is effective and safe and has outcomes comparable to wire localization, but with significant logistical benefit.

## Introduction

An increase in screen-detected breast cancer diagnoses has led to an increase in the need for breast lesion localization to allow intraoperative surgical guidance during breast-conserving surgery^1^.Wire-guided localization (WGL) has historically been the most common method of breast localization ^2^, along with ultrasound localization, radioactive seed localization, radio occult lesion localization, and tattooing. WGL has several potential limitations including difficulty sensing the end of the wire, dislodgement before surgery, and logistical restrictions due to placement of the wire immediately before surgery^3^.

New devices have been developed to try to improve the logistics of lesion localization and to uncouple localization from the date of surgery. The use of these devices has increased in recent years^4^ with multiple reasons considered by users including risk of device dislodgement, signal focus, and ease of logistics. These devices use a variety of different technologies to localize breast cancers, including paramagnetic (Magseed®, Endomagnetics Inc., Cambridge, UK)^5^ and permanently magnetic seeds (Pintuition®, Sirius Medical, Eindhoven, Netherlands)^6^, wireless radio frequency identification tags (LOCalizer™, Hologic, Santa Carla CA, USA)^7^, magnetic resonance imaging (MRI) guidance (Breast Cancer Locator™, Cairn Surgical, Lebanon, NH, USA)^8^ or SmartClip® technology (EnVisio Surgical Navigation System, Elucent Medical, MN, USA)^9^.

SCOUT® (Merit, South Jordan, UT, USA) is a radar reflector-based localization system that is licensed for use in breast and in axillary nodal localizations^10^. It can be placed at the time of biopsy^11^ or at any point before surgery^12^. The SCOUT® system is a 12 × 1.6 mm electromagnetic wave reflector that is inserted into the target lesion using a 16G introducer needle. The reflector is activated by infrared light emitted by the console probe and uses two antennas to reflect an electromagnetic wave signal back to the detector in the probe. Emerging data, mainly from single-centre studies, suggest that SCOUT® is effective in localizing lesions with outcomes comparable to WGL^13,14^ and demonstrating cost effectiveness^15^. Several groups have performed single institutional retrospective studies^16–19^ comparing the outcomes of SCOUT® *versus* WGL, but these are limited by their retrospective design, limited and heterogeneous definitions of study outcomes, and single-centre nature of the studies. Pooled analysis demonstrated statistically significant results for lower re-excision rates when SCOUT® was compared with WGL (8.6% *versus* 18.8%; *P* = 0.0001); however, this was based on a single institutional retrospective cohort study^20^ with a risk of bias.

Although randomized clinical trials are ideal, these are challenging in the context of breast lesion localization. The technique needs to be stable and standardized and needs to have been adopted by sufficient numbers of surgeons. Efficacy data are also required, and, given the potential for small differences between devices, large sample sizes are required so studies can be adequately powered. The iBRA-NET (implant Breast Reconstruction evaluation-NETwork) localization study is a well powered multicentre observational platform study designed to meet these needs and providing an alternative approach to traditional randomized trials. It uses the IDEAL (idea, development, exploration, assessment, long-term study)^21^ 2a/2b (development/exploration) framework to enable comparison of new localization devices with the standard of wire localization.

The iBRA-NET localization study has already reported on the outcomes of paramagnetic seed (Magseed®)^22^ and radiofrequency identification tag (LOCalizer™) localization^22,23^. In this fourth arm of the platform study, the results of two comparative arms of the study that investigate the outcomes of WGL and SCOUT® radar reflectors are reported.

## Methods

### Study design and participants

The iBRA-net localization study is a UK-based, international, multicentre, prospective, IDEAL stage 2a/2b platform cohort study, with embedded novel shared learning methodology^24^. This study compares the safety and effectiveness of radar-guided *versus* wire-guided breast lesion localization. The shared learning results will be reported elsewhere.

All UK surgical units performing wire or radar breast localization were invited to participate in the study through the following national professional organizations: Association of Breast Surgery, National Trainee Research Collaborative Network and Mammary Fold Research Network, and the iBRA-NET network of surgeons. International users of radar localization were also invited to contribute cases. Consecutive recruitment of women aged 16 and over, who had breast-conserving surgery requiring a preoperative localization procedure with radar between 1 June 2021 and 1 January 2025 was performed. The comparator historical cohort consisted of patients having WGL between August 2018 and August 2020. Ethical approval was not required as, per the Health Research Authority decision tool (http://www.hra-decisiontools.org.uk/research/), this was a service evaluation. Each participating centre was required to obtain local audit approvals and register the study before commencing recruitment, consistent with methods of previously reported multicentre prospective trainee collaborative studies^25,26^. Patient consent was not required for routine clinical data collection. Study data were collected in an anonymized format and managed using REDCap electronic data capture tools hosted at Manchester University NHS Foundation Trust^27^. The study involved the collection of clinical outcome data as routinely recommended by UK guidelines for good practice and outcomes were assessed against their quality standards^28,29^.

### Procedures

Study centres recruited consecutive patients undergoing breast localization procedures for breast or axillary localization, with the method of lesion localization (SCOUT® radar *versus* wire) depending on local availability and policy. Units recruited into either one (if only performing WGL or having switched to radar) or both arms of the study depending on local localization practice. Centres offering radar localization were provided with patient information leaflets. However, as this was a service evaluation, participating units were able to perform the procedures according to their routine practice. WGL was performed on the morning of surgery in all cases, whereas radar localization could be performed in advance.

### Quality assurance

For WGL surgery, the operating surgeon was expected to have completed a minimum of ten wire-guided wide local excisions in the last year. There was no requirement in the study to record radiology experience of device insertion. For radar localization surgery, participating units should have adopted the radar as their main method of localization and not be within the trial/early learning curve phase of the device (5 cases per surgeon). This was to ensure adequate expertise in both radiological placement and surgical removal of the radar. Individual surgeons must have completed a minimum of five successful radar localization cases and have completed their local training requirements before recruiting to the study.

### Outcomes

The primary outcome was effectiveness, defined as the identification and successful surgical removal or partial removal of the index cancer lesion or clip in the primary operation (confirmed as being present in the histopathological report). Secondary outcomes were safety (proportion of patients having a perioperative and postoperative complication, and proportion of those related to the device); close margins (where available, distance from nearest margin); weight of wide local excision specimen (g); breast reoperation rate (planned, which includes margin re-excision, and unplanned); cancellation rate on day of surgery (proportion of patients cancelled within 24 hours of time of surgery, and reason for the cancellation); and duration and start time of the surgical procedure. To account for lesion size in the assessment of excision weight, the weight/size^2^ is reported, in g/mm^2^. Other secondary outcome measures were the size of MRI bloom (in cases where MRI was performed with the SCOUT® reflector *in situ*), identification rate of axillary lymph nodes, timing of placement of the radar device (and proportion placed at time of biopsy), and transcutaneous detection rate^24^.

### Comparisons of radar localization *versus* wire—inclusion and exclusion criteria

**Figure 1:**
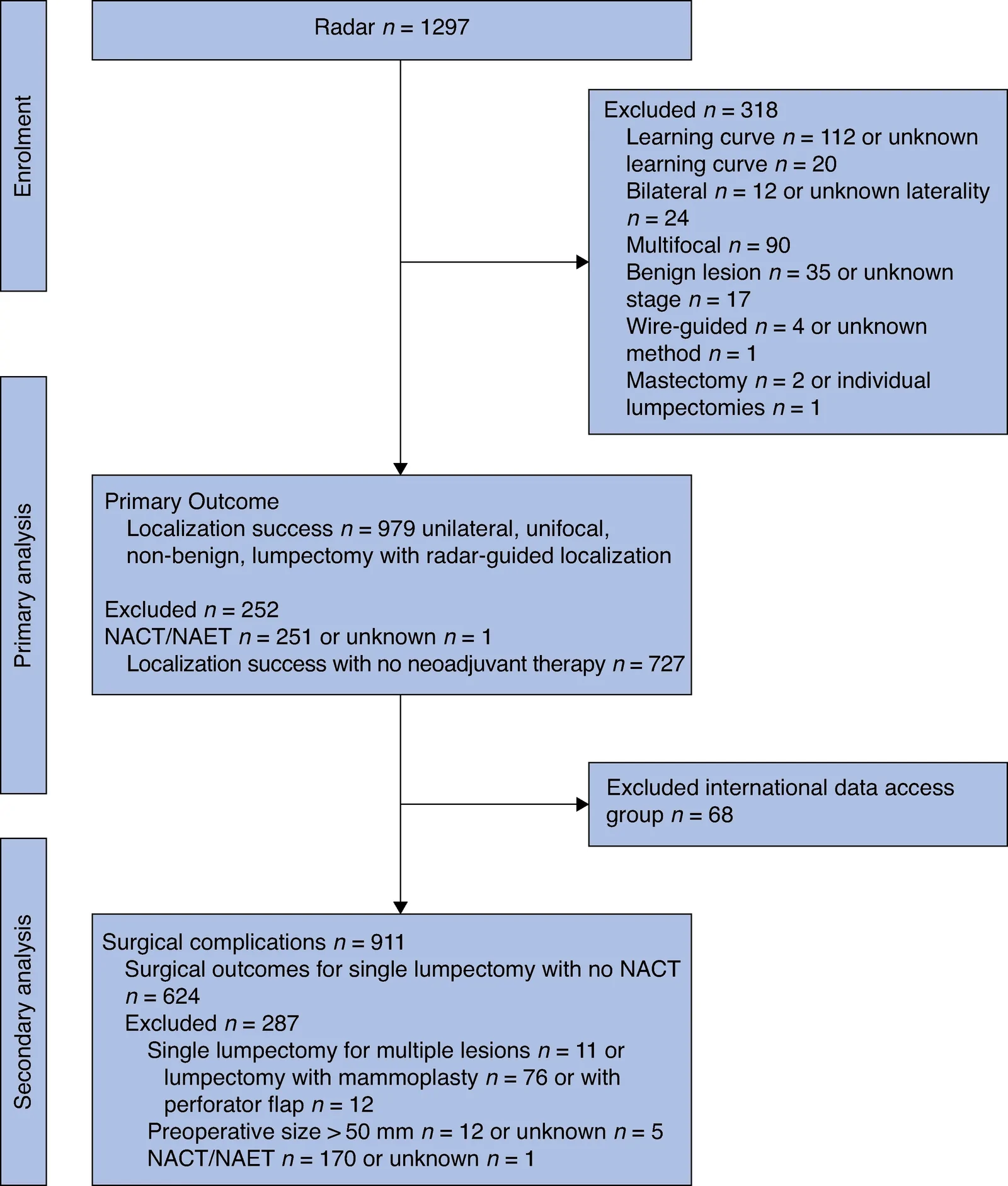
CONSORT flow diagram of radar localization patients Cases for analysis excluded those localizations performed in the learning curve or for bilateral or multifocal surgery. Secondary outcome measures—to enable for direct comparison of surgical outcomes between UK wire-guided localization and UK radar localizations international case entries were removed before analysis. NACT, neoadjuvant chemotherapy; NAET, neoadjuvant endocrine therapy.

Data were collected for all patients having a preoperative localization with a radar device to ensure all devices had safety data submitted and to provide a record for individual units of all the devices they had inserted during the audit period. Descriptive statistics were collected for these patients. Patients were excluded from primary analysis if the device was inserted in the learning curve period or unknown, if the localization was bilateral, multifocal, or benign. Patients were also excluded if they underwent mastectomy surgery. Primary analysis on this population looked at effectiveness of the device, safety, cancellation rate, MRI bloom size, and timing of placement and surgery. Only patients having unifocal, unilateral lesion localization performed in UK units were included in comparative secondary analysis of margin status, re-excision rates, and size of specimen to provide a better comparator to the WGL cohort, which is purely a UK cohort, with similar UK guidance for management and re-excision. For comparison of surgical parameters, only patients having a lumpectomy for a single unifocal tumour < 50 mm in size (cT1 and cT2 only) were included. If localization was stereo-guided, then preoperative size on mammography was used, and, if localization was ultrasound-guided, then size on ultrasound was used. Patients having two separate lumpectomies, those with larger tumours having a therapeutic mammoplasty, or volume replacement with lipofilling or perforator flap were therefore excluded from the analysis (see *Fig. 1*).

### Statistical analysis

The failure rate of WGL in the literature^30^is 0.6%, giving an identification rate of 99.4%. A clinically significant difference between techniques was considered to be less than 0.9%, assuming the two methods both have an expected identification rate of 99.4%^30^. To calculate the sample size, a power calculation was employed; the upper limit of the observed one-sided 95% confidence interval for the difference between identification rates (SCOUT® *versus* wire) is expected to be less than 0.9% with 80% power, requiring 1000 patients per group.

Descriptive statistics are reported for each outcome, as mean (standard deviation (s.d.)) or median (interquartile range (i.q.r.)), and as counts and percentages. Groups were compared using *t* tests, Wilcoxon rank-sum tests and χ^2^ tests, or Fisher’s exact test, as appropriate. All tests were two-sided with a significance level of 5%. Analyses were conducted using Stata® IC version 14 (StataCorp, College Station, TX, USA)^31^ and R version 4.4.1.

## Results

There were 1297 patients having radar-guided localization at 25 UK breast units and 2 international units (Chris O’Brien Lifehouse, Australia, and Hospital Universitario La Paz, Spain) between June 2021 and December 2024. Of these, 12 patients had bilateral localization and 11 patients had unilateral multifocal/multicentric localization and were excluded from the analysis (see *Fig. 1*). Incomplete records and records of wire-guided procedures submitted as controls for individual unit audit processes were also excluded. Patients undergoing surgery during the unit’s learning curve phase or when the learning curve data was missing were excluded from the analysis (132). The final cohort in the radar group therefore consisted of 979 patients. There were 1170 patients in the WGL comparator group from the original cohort, which has previously been reported.

### Primary endpoint

Patients within the two groups were comparable with respect to age and BMI (*Table 1*). There were differences in lesion size on preoperative imaging (*P* < 0.001), and there were more patients with ductal carcinoma *in situ* only in the WGL group compared with the radar-guided excisions (263 of 1170, 22.5% *versus* 158 of 979, 16.1%; *P* < 0.001). There were also differences in use of neoadjuvant chemotherapy (*P* < 0.001), surgical procedure (*P* < 0.001), and type of axillary surgery performed (*P* < 0.001).

**Table 1:** Clinicopathological variables in patients with localized breast lesions

	Wire *n* = 1170	Radar *n* = 979	*P*
Age (years), mean(s.d.)	60.4(10.6)	61(10)	0.178†
**BMI (kg/m^2^), median [i.q.r.]**	28 (24–32) [16–64]	28 (24.8–32.8) [17–68]	0.151‡
Unknown	235	217	
**Lesion size**	*n* **=** 931		
Mammogram (mm), median [i.q.r.]	13 (9–20)	15 (10–22) [3–73]	< 0.001‡
Unknown	75	92	
Ultrasound (mm), median [i.q.r.]	12 (8–17) [2–70]	13 (9–20) [2–79]	< 0.001‡
Unknown	239	113	
**Tumour stage**	*n* **=** 1147	*n* **=** 979	< 0.001
Tis	256 (22.3%)	133 (13.6%)	
T1	637 (55.5%)	552 (56.4%)	
T2	172 (15.0%)	226 (23.1%)	
T3	16 (1.4%)	21 (2.1%)	
yT0	66 (5.8%)	47 (4.8%)	
Unknown	23	-	
**Nodal stage**	*n* **=** 1139	*n* **=** 973	0.006
N0	973 (85.4%)	773 (79.4%)	
N1	105 (9.2%)	119 (12.2%)	
N2	11 (1.0%)	14 (1.4%)	
N3	8 (0.7%)	15 (1.5%)	
yN0	42 (3.7%)	52 (5.3%)	
Unknown	31	6	
**Histological diagnosis**	*n* **=** 1169	*n* **=** 979	< 0.001
Invasive ductal and lobular	716 (61.2%)	658 (67.2%)	
DCIS	263 (22.5%)	158 (16.1%)	
Mixed invasive/DCIS	127 (10.9%)	136 (13.9%)	
Other	63 (5.4%)	27 (2.8%)	
Unknown	1	-	
**Neoadjuvant therapy**	*n* **=** 1170	*n* **=** 978	< 0.001
Chemotherapy	117 (10.0%)	196 (20.0%)	
Endocrine therapy	50 (4.3%)	55 (5.6%)	
None	1003 (85.7%)	727 (84.3%)	
Unknown	0	1	
**Surgery**	*n* **=** 1170	*n* **=** 979	0.028
Lumpectomy for single lesion	1009 (86.2%)	870 (88.9%)	
Lumpectomy for multiple lesions	8 (0.7%)	11 (1.1%)	
Mammoplasty involving skin/volume reduction	142 (12.1%)	84 (8.6%)	
Perforator flap/lipofilling	11 (0.9%)	14 (1.4%)	
**Concurrent axillary surgery**	*n* **=** 1168	*n* **=** 979	< 0.001§
No	328 (28.1%)	145 (14.8%)	
Sentinel node biopsy	784 (67.1%)	761 (77.7%)	
Axillary clearance	56 (4.8%)	45 (4.6%)	
Targeted axillary dissection	-	28 (2.9%)	
Unknown	2	-	

Values are *n* (%) unless otherwise stated. s.d., standard deviation; i.q.r., interquartile range; DCIS, ductal carcinoma *in situ*. χ^2^ test, except †student’s *t* test, ‡Mann–Whitney U test, §Fisher’s exact test.

For radar-guided excisions (excluding benign disease, 35), the lesion was present in the excision specimen in 930 of 977 cases (95.2%), and for WGL (excluding benign disease) in 1150 of 1161 cases (99.1%) (*P* = 0.001) (*Table 2*). In 47 radar-guided cases and 11 WGL cases, the index lesion was classified as not removed in the excision specimen. In the post-neoadjuvant setting, a false negative value is presented in the event of a complete pathological response, where there is no index lesion remaining but the localization is likely to have been successful. Given the potential misclassification bias, a sensitivity analysis excluding patients receiving neoadjuvant chemotherapy was performed (*Table 2*). In this subgroup, identification rates were comparable with 99.6% (723 of 727) for radar and 98.9% (984 of 1003) for WGL (*P* = 0.114). *Table 2* demonstrates the primary endpoint data with patients having neoadjuvant chemotherapy removed, which more accurately reflects accuracy of target lesion removal.

**Table 2:** Primary endpoint: localization success of wire- and radar-guided localization

	Wire			Radar			P*
	Stereo	US	Total	Stereo	US	Total	
**All eligible patients**†	*n* = 337	*n* = 833	*n* = 1170	*n* = 139	*n* = 840	*n* = 979	
Successful localization							
Yes	325 (97.6%)	825 (99.6%)	1150 (99.1%)	132 (95.0%)	798 (95.2%)	930 (95.2%)	
No	8 (2.4%)	3 (0.4%)	11 (0.9%)	7 (5.0%)	40 (4.8%)	47 (4.8%)	< 0.001
Unknown	4	5	9	0	2	2	
**Sensitivity analysis, excluding patients who had neoadjuvant chemotherapy**‡	*n* = 296	*n* = 707	*n* = 1003	*n* = 122	*n* = 605	*n* = 727	
Successful localization							
Yes	284 (97.3%)	700 (99.6%)	984 (98.9%)	121 (99.2%)	602 (99.7%)	723 (99.6%)	
No	8 (2.7%)	3 (0.4%)	11 (1.1%)	1 (0.8%)	2 (0.3%)	3 (0.4%)	0.114
Unknown	4	4	8	-	1	1	

Values are *n* (%) unless otherwise stated. †The primary endpoint of localization success in all eligible patients; 44 of 47 patients recorded as ‘Successful localization —NO’ in the radar arm had recorded an outcome of ‘No index lesion present as there is a pCR’. ‡Excludes patients who had received neoadjuvant chemotherapy as many of these patients had a complete pathological response, some of these were incorrectly coded by the host units as the ‘target lesion not removed’. Stereo, stereotactic localization; US, ultrasound localization. *χ^2^ test, comparing ‘total wire’ *versus* ‘total radar’.

### Secondary endpoints

Surgical outcomes following lumpectomy for cT1/cT2 unifocal, unilateral lesions guided by radar and wire are shown in *Table 3*. Patients having neoadjuvant chemotherapy were excluded from this analysis. Positive margins (0 mm at ink) were seen in the main excision specimen in 9.0% of radar-guided *versus* 15.0% of WGL excisions (*P* < 0.001); however, the re-excision rates were similar at 14.7% *versus* 13.2% (*P* = 0.442). The groups were comparable for the rate of routine shaves taken at surgery and the duration of surgery. The specimen weight/size^2^ was higher in the radar cohort compared with WGL (0.182 g/mm^2^  *versus* 0.138 g/mm^2^; *P* < 0.001).

**Table 3:** **Surgical outcomes following lumpectomy for cT1/cT2 unifocal, unilateral lesions guided by radar and wire, excluding patients undergoing neoadjuvant chemotherapy**.

	Wire			Radar			
	Stereo *n* = 266	US *n* = 737	Total *n* = 1003	Stereo *n* = 87	US *n* = 537	Total *n* = 624	P*
**Margins**	*n* **=** 254	*n* **=** 720	*n* **=** 974	*n* **=** 87	*n* **=** 533	*n* **=** 620	
Positive (0 mm at ink)	43 (16.8%)	103 (14.3%)	146 (15.0%)	2 (2%)	54 (10.1%)	56 (9.0%)	< 0.001†
Unknown	12	17	29	0	4	4	
Closest margin (mm), median (range)	2 (0–20)	2 (0–35)	2 (0–35)	2.5 (0–12)	2 (0–88)	2.1 (0–88)	0.006
**Orientation of closest margin**	*n* **=** 252	*n* **=** 707	*n* **=** 959	*n* **=** 87	*n* **=** 532	*n* **=** 619	0.010†
Anterior	53 (21.0%)	192 (27.2%)	245 (25.3%)	19 (22%)	154 (29.0%)	173 (28.0%)	
Posterior	49 (19.4%)	146 (20.7%)	195 (20.5%)	20 (23%)	137 (25.8%)	157 (25.4%)	
Radial	150 (59.5%)	369 (52.2%)	519 (54.2%)	48 (55%)	241 (45.3%)	289 (46.7%)	
Unknown	14	30	44	0	5	5	
**Re-excision**	*n* **=** 260	*n* **=** 730	*n* **=** 990	*n* **=** 87	*n* **=** 531	*n* **=** 618	
Required	44 (16.9%)	87 (11.9%)	131 (13.2%)	16 (18%)	75 (14.1%)	91 (14.7%)	0.442†
Unknown	6	7	13	0	6	6	
**Of which, residual disease presents**	*n* **=** 42	*n* **=** 82	*n* **=** 124	*n* **=** 16	*n* **=** 72	*n* **=** 88	
Yes	15 (36%)	26 (32%)	41 (33.1%)	5 (31%)	29 (40%)	34 (39%)	0.490†
Unknown	2	5	7	0	3	3	
**Routine shaves/margins taken during surgery?**	*n* = 266	*n* = 737	*n* = 1003	*n* = 87	*n* = 537	*n* = 624	
No	76 (28.6%)	280 (38.0%)	356 (35.5%)	30 (35%)	197 (36.7%)	227 (36.4%)	0.758†
**Orientation of shave/margin taken**‡	*n* **=** 190	*n* **=** 457	*n* **=** 647	*n* **=** 57	*n* **=** 340	*n* **=** 397	
Anterior	13 (6.8%)	28 (6.1%)	41 (6.3%)	4 (7%)	17 (5.0%)	21 (5.3%)	0.575†
Posterior	39 (20.5%)	79 (17.3%)	118 (18.2%)	8 (14%)	44 (12.9%)	52 (13.1%)	0.036†
Radial	175 (92.1%)	431 (94.3%)	606 (93.7%)	52 (91%)	319 (93.8%)	371 (93.4%)	0.995†
**Duration of procedure**	*n* **=** 261	*n* **=** 728	*n* **=** 989	*n* **=** 59	*n* **=** 239	*n* **=** 298	
Duration (min), median (i.q.r.)	60 (45–75)	60 (45–85)	60 (45–82)	55 (43–70.5)	65 (51–84)	62.5 (48–81.75)	0.213
Unknown	5	9	14	28	298	326	
**Specimen weight/size^2^**	*n* **=** 227	*n* **=** 675	*n* **=** 902	*n* **=** 87	*n* **=** 534	*n* **=** 621	
Specimen (g/mm^2^), median (i.q.r.)	0.133 (0.03–0.94)	0.14 (0.07–0.29)	0.138 (0.06–0.32)	0.2 (0.09–0.5)	0.181 (0.1–0.37)	0.182 (0.1–0.38)	< 0.001
Unknown	39	62	101	0	3	3	

Values are *n* (%) unless otherwise stated. ‡Not mutually exclusive, so for each patient, multiple shaves have been taken. min, minutes; i.q.r., interquartile range. *Mann–Whitney U test, except †χ^2^ test.

In the majority of circumstances (94.6%), localization was performed before the day of surgery in the radar group. A minority (43 of 228, 18.9%) of the radar devices were placed at the time of biopsy. There was no difference in time taken to complete the surgery (median 62.5 minutes (min) *versus* 60 min; *P* = 0.213); however, the groups were not matched according to concurrent axillary surgery being performed, with more axillary surgery being performed in the radar *versus* WGL group, with 85.2% of radar procedures having concurrent axillary surgery *versus* 71.9% WGL procedures (*Table 1*). During the perioperative procedure (*Table 4*), there was no difference in the rate of self-reporting of the localization device becoming dislodged from the lesion in WGL (1.4%) *versus* radar-guided (0.5%) surgery (*P* = 0.066). The radar device was undetectable percutaneously in 6 (0.7%) cases, but all procedures were performed as planned with the lesions safely removed without further localization. There was no difference in all postoperative complications (*P* = 0.070) or in complications directly related to localization device (*P* = 0.660) (*Table 4*). There was a higher rate of reoperation within 30 days in the WGL group compared with the radar group (4.6% *versus* 1.5%; *P* < 0.001), although there was no difference in unexpected readmission to hospital within 30 days (*P* = 0.938). Reoperations were reported in both groups for similar reasons: margins being close or needing re-excision, for further axillary surgery (sentinel lymph node biopsy or axillary lymph node dissection), for evacuation of haematomas, and one case of wound dehiscence in the SCOUT® arm.

**Table 4:** Surgical complications in radar- and wire-guided surgery for unifocal, unilateral lesions

	Wire	Radar	Total	P*
**Perioperative localization-related complications**	*n* **=** 1157	*n* **=** 829	*n* **=** 1986	
None	1124 (97.1%)	813 (98.1%)	1937 (97.5%)	0.246
Dislodged from lesion	16 (1.4%)	4 (0.5%)	20 (1.0%)	0.066†
Not detectable percutaneously	-	6 (0.7%)	6 (0.3%)	0.005
Index lesion/clip not visible on specimen radiographs	9 (0.8%)	1 (0.1%)	10 (0.5%)	0.053†
Procedure abandoned, further localization required	-	-	-	-
Other	11 (1.0%)	6 (0.7%)	17 (0.9%)	0.632†
**Postoperative surgery-related complications**	*n* **=** 1149	*n* **=** 907	*n* **=** 2056	
None	1092 (95%)	844 (93.1%)	1936 (94.2%)	0.070
Seroma requiring aspiration	55 (4.8%)	62 (6.8%)	117 (5.7%)	0.058
Haematoma requiring aspiration in clinic	16 (1.4%)	13 (1.4%)	29 (1.4%)	1.000†
Haematoma requiring surgical evacuation	7 (0.6%)	9 (1%)	16 (0.8%)	0.466†
Minor wound infection (oral antibiotics)	54 (4.7%)	28 (3.1%)	82 (4%)	0.082
Major wound infection (intravenous antibiotics)	11 (1%)	3 (0.3%)	14 (0.7%)	0.108†
Major wound infection (drainage/debridement)	6 (0.5%)	4 (0.4%)	10 (0.5%)	1.000†
**Postoperative complications (general)**	*n* **=** 1152	*n* **=** 908	*n* **=** 2060	
In-hospital complication including systemic complications such as DVT/MI/PE	4 (0.3%)	2 (0.2%)	6 (0.3%)	0.700†
Unexpected readmission to hospital within 30 days	11 (1%)	9 (1%)	20 (1%)	1.000†
Readmission directly related to the localization procedure	3 (0.3%)	0	3 (0.1%)	0.260†
Reoperation within 30 days	53 (4.6%)	14 (1.5%)	67 (3.3%)	< 0.001
Complications directly related to localization device	2 (0.2%)	3 (0.3%)	5 (0.2%)	0.660†

Values are *n* (%) unless otherwise stated. DVT, deep venous thrombosis; MI, myocardial infarction; PE, pulmonary embolism. *χ^2^ test, except †Fisher’s exact test.

Operations were able to be performed significantly earlier in the day in the radar cohort (459) *versus* WGL (1124), with a median start time of 12:27 *versus* 13:40 (*P* < 0.001), with 23.6% of radar cases performed first on the list. Three (0.3%) patients in the WGL group and eight (0.8%) patients in the radar group had their surgery cancelled on the day (*P* = 0.075), and one patient in each group was related to a problem with the localization.

MRI bloom size in the radar group (*n* = 33) was a median of 11 (i.q.r. 8–13) mm.

### Axillary placement

There were 18 patients that had radar localization of the axillary lymph node, with the majority of patients having placement in a single node (14 of 18, 4 unknown). Of those nodes clipped before neoadjuvant chemotherapy (9), two were marked with a titanium clip and seven with a radar device. The radar device and index lymph node were retrieved together in all recorded cases (13).

## Discussion

This study reports on the radar arm of the iBRA-NET localization studies, the largest prospective multicentre study to date comparing WGL breast surgery and novel non-wire, non-radioactive, non-magnetic localization device-guided surgery. The primary analysis showed significantly higher localization rates in the wire group, but the sensitivity analysis showed comparable rates, excluding patients undergoing neoadjuvant chemotherapy. The localization rate was high in both of the two cohorts, with an accuracy of 99.6% in the radar group. The primary outcome measure for this study was user-reported ‘Identification and successful surgical removal or partial removal of the index lesion in the primary operation’, the index lesion being defined as either tumour or the clip marking the tumour if there was no residual tumour. Using user responses only, the radar device had an accuracy of 95.2%, in 4.8% of cases it was analysed that the target lesion was not removed. In 44 records, the response to the primary outcome was ‘No index lesion present as there is a pCR’ (pathological complete response). In previous arms of the study, this was analysed as ‘Index lesion not removed’, but, as this was a rarely recorded outcome, it therefore did not have a large effect on the localization accuracy. Although 44 cases had this outcome, this was not necessarily a failure of localization as the index lesion may well have been successfully removed; the patient just had a pCR. Because in the other arms of the study this was recorded as a failure of localization, the same method of analysis has been maintained. The sensitivity analysis therefore represents a more accurate representation of the localization accuracy. If those 44 patients were analysed as undergoing successful localization of the pCR lesion, then the primary analysis would have shown an accuracy of 99.7% in the radar arm. To explore the impact of this potential misclassification, a sensitivity analysis excluding patients receiving neoadjuvant chemotherapy was performed.

Re-excision rates for involved or close margins were 14.7% in this series and not significantly different from the WGL arm, despite the positive margin rate (0 mm at ink) being significantly higher in the WGL cohort compared with the radar cohort (15% *versus* 9%). The reason why the higher positive margin rate in the WGL cohort did not translate into a higher re-excision rate is likely to be multifactorial, which may include the use of shaves and the local guidance on margin width and management. A secondary analysis of margin assessment, to include all arms of the iBRANET localization study, is planned and may help elucidate the causation of these differences. Tingen *et al*.^17^, in a smaller cohort of patients from a single institution, compared radar with WGL, and their re-excision rates were 5.3% radar *versus* 13.7% WGL. Pooled analysis of 842 reflectors in eleven studies demonstrated a re-excision rate of 12.9% in the radar localized group, which is broadly similar to the present findings^32^. This data set has the advantage of well defined outcome measures and being multicentre, multiuser, post-device learning, which should make the findings more applicable to the wider surgical community. The findings from all arms of the localization study thus far do not demonstrate a reduction in re-excision rate for any of the three devices (Magseed®, LOCalizer™, SCOUT®) compared with WGL^22,23^. This may suggest that margin involvement is determined more by pathological assessment and disease extent rather than localization technique.

This study also found that the specimen weight/size^2^ was higher in the radar cohort compared with WGL (0.182 g/mm^2^  *versus* 0.138 g/mm^2^; *P* < 0.001); this is in contrast to the other arms of the localization study, which showed no significant difference between WGL and paramagnetic seeds and wireless radio frequency identification tags. One reason for recording specimen weight/size^2^ is that it was a commonly held marketing strategy that these devices would result in smaller specimens; however, the iBRAnet localization studies have never shown a reduction in specimen size with the newer generation devices. It is unclear why, but these findings suggest that it does not give surgeons an advantage in terms of reducing specimen size. There were 621 localizations in the analysis for specimen size, and this result would need to be confirmed in another study as there are potential confounders between the two groups. This may lend itself to a randomized study with stratification, so that the two arms of the randomized control study are truly balanced.

This study also aimed to assess the efficacy of the radar device in successfully localizing axillary lymph nodes. All nodes were successfully localized with the device; however, there was no control group in this outcome of the study. The 100% accuracy in the present study adds to the emerging body of evidence on its efficacy in this setting, with a recent meta-analysis demonstrating 99.6% accuracy in localization in a total of 252 procedures^13^. This suggests the device can be used localize axillary lymph nodes, but the numbers assessed in this study are small.

One possible advantage of this device is the potential to cause a smaller bloom/artefact on an MRI compared with other wire-free devices. Bloom size will be a combination of the bloom created by the radar device and any other clips present in the lesion. A smaller MRI bloom means less of a dead spot on an MRI scan of the breast for a patient with an implanted radar device. MRI bloom size was on average 11 mm in the iBRA-NET study, which compares to < 5 mm in a previous study of six patients^33^. This means the MRI artefact will have a clinical impact on the imaging appearances of the index lesion, but would be diagnostic in other areas of the breast/axilla.

Due to its minimal interference on MRI, and its licencing for indefinite implantation without the need for surgical excision, SCOUT® can potentially be placed at the time of biopsy if imaging is felt to be diagnostic of cancer. Logistically, this saves the patient having a second procedure of a localization and can be placed in lesions with 98% certainty they will ultimately be malignant, elevated risk, or discordant^11^. In this iBRA-NET cohort, the radar device was placed at the time of biopsy in 42 procedures. All of these were malignant; however, this study was not designed to evaluate the efficacy or logistical benefits of this approach, but nevertheless appears feasible.

Medical device safety means identifying risks, preventing harm, and ensuring reliable performance. This study confirms the reliability of the device in efficacy of localization and demonstrates comparable results in re-excision of margins. This study demonstrates the risk of non-detection of the device percutaneously and gives us an incidence of this hazard (0.7%), and also suggests that this is unlikely to cause consequences for the patient, as the lesions were all removed at time of operation. There was no statistical difference in the rate of device dislodgement between wires and radar devices (1.4% *versus* 0.5%), but the present study may be underpowered to find a difference and, given the retrospective nature of the study, these data are reliant on self-reporting accuracy. Device dislodgment was not defined in the question ‘Wire dislodged from lesion’ or ‘Scout dislodged from lesion’, which is a limitation to interpreting the outcomes as the reasons given for SCOUT® dislodgement include SCOUT® being deactivated during surgery, ‘Scout falling off specimen prior to X-ray’, and ‘Scout lying superior to lesion on intraoperative ultrasound so WGL placed’. In only one SCOUT® ‘dislodgement’ was another localization method required; the lesions were successfully removed in all other cases. Overall, the study demonstrates a similar safety profile to WGL.

This study has limitations. These mainly relate to the comparisons with a ‘historical cohort’, as certain components of practice, such as national recommendations regarding margin width and use of neoadjuvant chemotherapy, have changed such that the groups are not always directly comparable. There is also potential selection bias as units were not performing both radar and WGL excision, and surgeons and units have differences in disease presentation, preoperative imaging, and postoperative pathology assessment, all of which impact outcomes. The main primary outcome measure of outcome of localization is unlikely to be affected by a change in guidance or practice over time, and can be considered robust, but re-excision rate and specimen size do have the potential for confounder bias. These devices are placed when the user feels there is a need for a localization device, the groups are not completely matched by tumour stage, and it is possible that some of these lesions may have been partially palpable. Although it is unlikely that this would affect the overall conclusions of the study, this is an inherent limitation of a non-randomized study with a historical comparator. However, all units are exposed to the same national guidance and standards, and one strength of the study is the large number of contributing units and localizations, which help to minimize bias.

The study was predesigned with a historical cohort of WGL patients, the drawback being for the newer study arms it becomes more historical, and the advantage being that it enables fast and efficient addition of new device arms and availability of data to users and patients. This study did not examine cost effectiveness, or clinician or patient satisfaction with either technique, although cost-effectiveness has been examined in another non-prospective study^15^.

The development of non-palpable breast lesion localization devices, such as radar reflectors, provides a safe alternative to wires, with potential logistical benefits, including uncoupling localization from the date of surgery. Further work will include patient and clinician preference outcomes, and a cost-analysis comparison to evaluate fully these technologies, as well as an assessment of their ability to localize axillary lymph nodes accurately to facilitate targeted axillary dissection. Ongoing collaboration with European colleagues has led to the development of the MELODY study^34^, which will report on several localization devices, and includes patient-reported outcomes.

## Data Availability

Data are not publicly available.

## Collaborators

Members of the iBRA-NET Localisation Study collaborative: A. Pradeep, S. Muktar (Addenbrookes Hospital, Cambridge, UK); L. Martin, J. Noronha, K. Mallalieu (Aintree University Hospital, Aintree, UK); S. Ledwidge, J. Tan, S. Khan (Barts Health NHS Trust, London, UK); D. Debnath, P. McAleese, A. McKenna, E. Afify, L. Ballance (Blackpool Victoria Hospital, Blackpool, UK); P. O’Leary (Bon Secours, Cork, Ireland); F. Meybodi (Breast Cancer Institute, Sydney, Australia); I. Oikonomou, C. Liyang Wang, Mok (Cambridge University Hospitals NHS Foundation Trust, Cambridge, UK); C. Hollywood (Chesterfield Royal Hospital NHS Foundation Trust, Chesterfield, UK); C. Mak (Chris O'Brien Lifehouse, Sydney, Australia); S. Naz (County Durham and Darlington NHS Foundation Trust, Durham, UK); K. Kolar (Doncaster and Bassetlaw Teaching Hospitals NHS Foundation Trust, Doncaster, UK); C. Roshanlall (East Cheshire NHS Trust, Macclesfield, UK); R. Swan (Edinburgh Breast Unit, Edinburgh, UK); M. Gasparri (Ente Ospedaliero Cantonale, Bellinzona, Switzerland); M. Walker (Epworth, Melbourne, Australia); C. Ríos Gozálvez, C. Sitges, A. Catanese, C. Sitges, A. Mariscal (Hospital Germans Trias i Pujol, Barcelona, Spain); J. Sánchez-Méndez (Hospital Universitario La Paz, Madrid, Spain); E. Khalifa (Hull University Teaching Hospitals, Hull, UK); A. Nagarajakumar, I. Giono (King's College Hospital NHS Trust, London, UK); E. de Sousa, J. Henderson, L. Swainbank (Liverpool University Hospitals NHS Foundation Trust, Liverpool, UK); I. Sallam (MISR INTERNATIONAL HOSPITAL, Giza, Egypt); R. Lefroy (New Cross Hospital, Wolverhapmton, UK); G. Babu, E. Macaskill, K. McNeil (NHS Tayside, Dundee, UK); S. Vural, A. Aber, N. Emmanuel (Princess Royal University Hospital, London, UK); L. Gharbawi (Queen Alexandra Hospital, Portsmouth, UK); A. Taniskidi, J. Ragunathan (Queen Mary University of London, London, UK); K. Griffith (Rose Medical Center, Denver, USA); B. Smith, A. Hakim, A. Bassi (Royal Berkshire Hospital, Reading, UK); R. Burrows (Royal Derby Hospital, Derby, UK); L. Mackay, H. Fatayer, S. D’Costa, M. Abdelhadi, I. Whitehead, G. Ng (Royal Liverpool University Hospital NHS Foundation Trust, Liverpool, UK); S. Mylvaganam, E. Rahman (Royal Wolverhampton Hospitals NHS Trust, Wolverhampton, UK); I. Sallam (Sheffield Teaching Hospitals, Sheffield, UK); M. Shaaban (South Tees NHS Foundation Trust, Middlesbrough, UK); A. Michaelides (St Bart's Hospital, London, UK); M. Khatib (Tel Aviv Medical Centre, Tel Aviv, Israel); N. Ditsch (Universitätsfrauenklinik Augsburg, Augsburg, Germany); A. Hamad (University Hospitals of Derby & Burton NHS Trust, Derby, UK); E. Mohamed (Whipps Cross Hospital, London, UK); O. Alaamer (Womens Health Hospital, National Guard Health Affairs, Riyadh, Saudi Arabia).
